# Humoral Immune Response to Keyhole Limpet Haemocyanin, the Protein Carrier in Cancer Vaccines

**DOI:** 10.1155/2011/614383

**Published:** 2011-06-12

**Authors:** A. Kantele, M. P. Häkkinen, J. Zivny, C. O. Elson, J. Mestecky, J. M. Kantele

**Affiliations:** ^1^Division of Infectious Diseases, Department of Medicine, Helsinki University Central Hospital, Aurora Hospital, Building 5, 3rd floor, P.O. Box 348, 00029 Helsinki, Finland; ^2^Department of Bacteriology and Immunology, Haartman Institute, FIN-00014 University of Helsinki, Finland; ^3^Departments of Microbiology and Medicine, The University of Alabama at Birmingham, Birmingham, AL 35294-2170, USA; ^4^Institute of Pathological Physiology, First Faculty of Medicine, Charles University in Prague, 12853 Prague 2, Czech Republic; ^5^The University of Alabama at Birmingham, Birmingham, AL 35294, USA; ^6^Department of Medical Microbiology and Immunology, University of Turku, FIN-20520 Turku, Finland

## Abstract

Keyhole limpet haemocyanin (KLH) appears to be a promising protein carrier for tumor antigens in numerous cancer vaccine candidates. The humoral immune response to KLH was characterized at the single-cell level with ELISPOT combined with separations of cell populations according to their expression of homing receptors (HRs). The analysis of HR expressions is expected to reveal the targeting of the immune response in the body. Eight orally primed and four nonprimed volunteers received KLH-vaccine subcutaneously. Circulating KLH-specific plasmablasts were found in all volunteers, 60 KLH-specific plasmablasts/10^6^ PBMC in the nonprimed and 136/10^6^ in the primed group. The proportion of L-selectin^+^ plasmablasts proved high and integrin **α**_4_ **β**_7_^+^ low. KLH serving as protein carrier in several vaccines, the homing profile of KLH-specific response may be applicable to the cancer antigen parts in the same vaccines. The present data reflect a systemic homing profile, which appears advantageous for the targeting of immune response to cancer vaccines.

## 1. Introduction

Many important antigens, whether of cancerous or microbial origin, are poorly immunogenic when injected into humans in a soluble form. However, it is possible to significantly improve their immunogenicity by conjugation to a highly immunogenic protein carrier such as tetanus toxoid (TT) or keyhole limpet haemocyanin (KLH) [1, 2]. This conjugation ensures that the robust T-cell help elicited by the carrier protein is concentrated in the vicinity of T- and B-cells specific to the weak antigen to which the protein carrier is linked. This in turn facilitates the T-B cell cooperation and results in a more vigorous immune response to the weak antigen [2]. This approach has proved highly successful in vaccines containing bacterial polysaccharides conjugated to a protein carrier [1]: pneumococcal and *Haemophilus influenzae* type b—TT conjugate vaccines, for example, are used widely and have been introduced into national vaccination programmes in several countries. Numerous cancer antigens have been linked with KLH, resulting in promising anticancer vaccine candidates [2–7].

KLH is a naturally occurring immunoadjuvant functioning as a respiratory protein of giant keyhole limpets living in shallow coastal waters in a sea [8, 9]. It is a mixture of two immunologically distinct isoforms, both of which are didecamers assembled from 400 kDa polypeptides; an atomistic model of the quaternary structure of isoform KLH1 is available [8]. This antigen is ordinarily not encountered by the human immune system. KLH has been used in studies addressing the phenomenon of oral tolerance; prolonged oral priming with soluble KLH has been shown to result in a suppression of T- cell-mediated immunity and delayed hypersensitivity reaction after a subsequent subcutaneous immunization [10, 11]. In contrast to this, the humoral immune response at both mucosal and systemic sites has proved to be enhanced [10]. These studies were carried out to provide tools for eliciting oral tolerance useful in numerous autoimmune diseases. 

In addition to the tolerance studies, KLH has been explored in cancer research. Originally, it was found to be cross-reactive with cancer antigens in urinary bladder carcinoma, which led to research into the use of KLH as bladder carcinoma vaccine [2, 9]. More recently, however, KLH has proved successful as a protein carrier to numerous poorly immunogenic cancer antigens such as in vaccines against follicular lymphoma [3], non-Hodgkin lymphoma [6], glioblastoma multiforme [4], melanoma [2], prostate, and ovarian cancer [2]. The conjugate cancer vaccines appear as the most extensively studied application of KLH at the moment.

Immune response to vaccination is not distributed evenly in the body, but, instead, activated lymphocyte populations are guided to traffic only to certain tissues [12, 13]. Tissue-specific migration is based on a multistep process of homing; while the blood carries the activated lymphocytes everywhere in the body, the cells can lodge into tissues only at sites which they recognize by means of their specific surface molecules [12, 13]. The most important molecules determining tissue localization are chemokine (CCR) and homing receptors (HRs) [12, 13], which recognize their ligands in the tissue, chemokines, and endothelial addressins, respectively. Several tissue-specific HRs have been identified; the intestinal HR, *α*_*4*_ *β*_*7*_ integrin, guides the cells to the intestinal mucosa [14], L-selectin (CD62L) to peripheral lymph nodes [15, 16] and cutaneous lymphocyte antigen (CLA) to cutaneous sites [17, 18]. We have shown earlier that KLH-specific T-cells after oral feeding express significantly more *α*_*4*_ *β*_*7*_ integrin than KLH-specific T-cells after parenteral injection. Thus, the homing profile of KLH-specific T cells depends on the site of antigen encounter [19]. With other antigens, the homing profile of B cells has been found to depend on the site of antigen encounter [20–23]. The homing of B cells after immunization with KLH has not been addressed in any studies before.

The present study characterized at a single-cell level the immune response to KLH and the homing profiles of these cells. KLH being a common protein carrier to numerous cancer antigens in various vaccines [2–7], this homing profile is of special interest, for it can be interpreted as the homing profile associated with all the different injectable cancer vaccines linked to KLH, and thus give an insight into the localization of the response to these vaccines. Optimal targeting of the ensuing immune response should always be one of the goals of vaccine development. As to cancer vaccines in general, it appears most beneficial to target the immune responses to the site where the primary tumor developed.

## 2. Materials and Methods

### 2.1. Volunteers

Fourteen healthy volunteers (8 women, 6 men, aged 22–40 years) participated in the study. None of the volunteers had been previously exposed to KLH; all of them had received TT vaccine according to the usual vaccination protocol as a child and a booster dose within the last ten years. The study was approved by the Human Use Committee of the University of Alabama at Birmingham. Informed consent was obtained from each volunteer before participation.

### 2.2. Experimental Design

**Figure 1:**
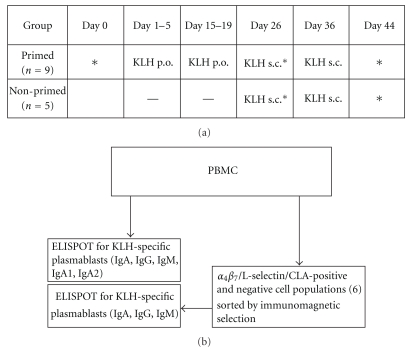
Experimental design of the study. (a) Protocol for immunization with KLH and for collection of blood samples. The asterisks indicate days of collecting blood samples. (b) Assaying KLH-specific circulating plasmablasts from the blood samples. The cells were assayed both from the total population of PBMC and from receptor-positive and -negative populations (a total of six separated populations for each volunteer) resulting from immunomagnetic sorting with respect to different HR.

Nine fasting volunteers ingested each 100 mg of KLH on days 1 to 5 and days 15 to 19 and were given 100 *μ*g KLH subcutaneously on days 26 and 36 (primed group) (Figure 1(a)). Another 5 volunteers received only the parenteral KLH on days 26 and 36 (nonprimed group). Blood samples were drawn on days 0, 9, and 44. Mononuclear cells were isolated from the blood samples and subjected to immunomagnetic cell selection to sort the cells into receptor-positive and-negative cell populations with respect to their expression of *α*_*4*_ *β*_*7*_ integrin and L-selectin (Figure 1(b)). These populations were assayed for KLH- specific ASC with enzyme-linked immunospot (ELISPOT) assay. Due to the limited numbers of cells obtained, all assays could not be carried out on all volunteers.

### 2.3. KLH

KLH as a freeze-dried powder was purchased from Calbiochem Corp. (La Jolla, CA, USA). For oral use, 100 mg of this preparation was packed into gelatin capsules. KLH for parenteral use was purchased from Pacific Biomarine (Venice, CA, USA). It was purified from an ammonium sulphate preparation of the protein. This preparation was dissolved in pyrogen-free saline, passed twice through a polymyxin-agarose column, and assayed for endotoxin content with Limulus assay as described in detail earlier [10].

### 2.4. Isolation of Mononuclear Cells

Mononuclear cells were isolated with Ficoll-Paque density-gradient centrifugation from heparinized venous blood. The cells were washed twice with PBS and suspended in culture medium (RPMI-1640 supplemented with 10% heat inactivated fetal calf serum, 100 U penicillin/mL and 100 *μ*g streptomycin/mL) [19].

### 2.5. Separation of the Receptor-Negative and -Positive Cell Populations

The separation of the cells into receptor-negative and -positive populations has been described in detail earlier [20, 22, 24]. Briefly, cells were incubated with one of the first-stage monoclonal antibodies: anti-*α*_4_ *β*_7_ (ACT-1; Millennium Pharmaceuticals, Cambridge, MA, USA) or anti-L-selectin (anti-CD62L; Becton-Dickinson, San Jose, CA, USA). After washing the cells were incubated with Dynal M-450 magnetic beads coated with sheep antimouse IgG (Dynal, Oslo). Receptor-positive and -negative cells were separated with magnetic cell sorting. Both the receptor-positive and -negative cell populations were immediately analyzed with the ELISPOT assay for numbers of all immunoglobulin-secreting cells (ISC) and KLH-specific ASC. The efficiency of the cell separations was checked with flow cytometry in pilot experiments as described earlier [20]; >90% of the L-selectin^+^ cells including all brightly staining cells and >99% of the integrin *α*_4_ *β*_7_^+^ cells were removed from the unsorted cells by the negative selection.

### 2.6. Assay of ISC and ASC (ELISPOT)

While ISC represents all plasmablasts in the circulation, antigen-specific plasmablasts represent smaller subpopulations within the whole population of ISC. KLH-specific plasmablasts were identified as ASC specific for KLH. In the ELISPOT assay, plasmablasts are allowed to secrete antibodies in the immediate vicinity of the antigen (capturing Ig in ISC assays and antigen in ASC assays) on a microwell plate, and the antibodies secreted are then detected immunoenzymatically. The substrate is added in hot agarose to immobilize the decaying colour into a spot. Each spot is regarded to correspond to a single-cell secreting antibodies specific to or captured by the coating antigen. 

The assays of IgA-, IgG- and IgM-ISC/ASC [25] and IgA1-and IgA2-ISC [26, 27] have been described in detail previously. Briefly, microtiter plates were coated with antisera to human IgA or IgM (Dako, Glostrup, Denmark) or IgG (Sigma, St. Louis, MO, USA) for the ISC-assay, or with KLH (5 *μ*g/mL PBS) for the ASC assay and blocked with 1% bovine serum albumin. The cells were incubated in the wells for 2-3 h, and antibodies secreted during this time were detected with alkaline phosphatase-conjugated antihuman IgA (Sigma-Aldrich), IgG (Sigma-Aldrich) and IgM (SouthernBiotech, Birmingham, AL, USA) antisera or, for the IgA-subclass assays, with monoclonal antibodies to IgA1 and IgA2 (Nordic Immunological Laboratories, Tilburg, the Netherlands) followed by alkaline phosphatase-conjugated antimouse IgG (Jackson ImmunoResearch Laboratories, West Grove, PA, USA). Substrate was added to all plates in hot agarose as described in detail earlier [25].

### 2.7. Statistics

Percentages of cells expressing the different receptors were determined as arithmetic means of the percentages of ISC expressing the given cell surface marker. The proportions of the receptor-positive ISC were calculated as follows: % of receptor-positive cells among ISC = (100 × the number of ASC or ISC in receptor-positive population) ÷ (the sum of the number of ASC or ISC in receptor-positive and receptor-negative populations). In order to get reliable statistics for the proportions of cells expressing a given marker, we set an inclusion limit of 20 ASC that needed to be identified among the cells studied. Statistical analyses were performed with Student's *t*-test, and the results were considered significant only when *P* < .05.

## 3. Results

### 3.1. Numbers of Total Ig-, IgA1-, and IgA2-Immunoglobulin-Secreting Cells (ISC)

The total populations of ISC represent the sum of numerous antigen-specific populations of ASC. On the average, 0.2% of peripheral blood mononuclear cells (PBMC) were ISC (plasmablasts). The geometric mean of IgA-ISC was 864, IgG-ISC 800 and IgM-ISC 89 cells/10^6^ PBMC; 75% of IgA-ISC were found to secrete IgA1 and 25% IgA2.

### 3.2. KLH-Specific IgA-, IgG-, IgM-, IgA1-, and IgA2-ASC

**Figure 2:**
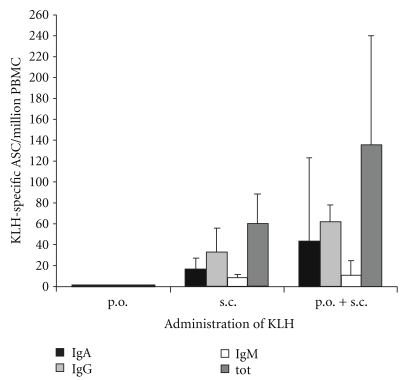
The numbers of KLH-specific plasmablasts identified with ELISPOT as KLH-specific antibody-secreting cells (ASC) in the circulation of vaccinees after oral KLH feeding (*n* = 9) or after two subcutaneous KLH injections of nonprimed (*n* = 5) or orally primed (*n* = 9) volunteers. The data are given as geometric means of ASC/10^6^ PBMC ± SEM.

Before immunizations, no KLH-specific ASCs were found in the circulation of any of the vaccinees. In the primed group, none of the 9 volunteers had a response after oral administration of KLH. After two subcutaneous injections, 9/9 of these volunteers had an ASC response with a geometric mean of 136 ASC/10^6^ PBMC (Figure 2). In the nonprimed group, 5/5 volunteers responded after two subcutaneous KLH injections with a geometric mean of 60 ASC/10^6^ PBMC (Figure 2). 

In orally primed volunteers, IgA predominated in 4/9 and IgG in 5/9 volunteers. In the nonprimed group, IgA predominated in 2/5 and IgG in 3/5 volunteers.

KLH-specific IgA1- and IgA2-ASC were determined in 2/5 and 6/9 volunteers in the orally primed and nonprimed groups, respectively. The mean percentage of KLH-specific IgA1-ASC was 76% and 86% in these volunteers, respectively.

### 3.3. The Expression of Homing Receptors on KLH-Specific IgA-, IgG-, and IgM-ASC after Parenteral Vaccination

**Figure 3:**
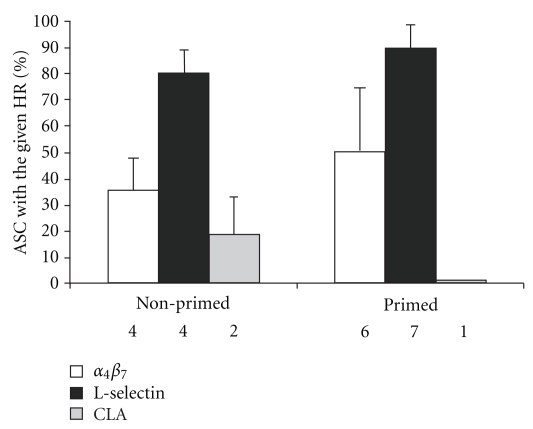
The expression of the intestinal homing receptor, *α*_4_ *β*_7_ integrin, the peripheral lymph node HR, L-selectin, and the skin HR, CLA, on KLH-specific plasmablasts after two subcutaneous KLH injections given to orally primed or nonprimed volunteers. The data were calculated by counting the proportion of HR positive cells among all cells (the sum of HR positive and negative cells). The data are given as arithmetic means of the percentage of ASC expressing the given receptor ± SD. The numbers of volunteers from whom the data were pooled are indicated under each bar.

The expressions of *α*_4_ *β*_7_ integrin, L-selectin, and CLA on circulating KLH-specific ASC are shown in Figure 3. L-selectin was found to be expressed more frequently than *α*_4_ *β*_7_ integrin on KLH-specific ASC (*P* < .001) in both the primed (*P* < .01) and nonprimed (*P* < .01) groups. Only 35% of KLH-specific ASC expressed *α*_4_ *β*_7_ integrin in the nonprimed group and 50% in the primed group (Figure 3). L-selectin was expressed by 80% and 89% of KLH-specific plasmablasts in nonprimed and primed groups, respectively. CLA expression was determined from only three volunteers (Figure 3). The results on *α*_4_ *β*_7_ integrin- and L-selectin- expressions indicate a nonintestinal, systemic homing profile.

No major differences were seen in the homing profiles between the cells secreting IgA, IgG, and IgM (data not shown).

The sum of percentages of cells expressing the various HR exceeded 100% in all patients with data available from both *α*_4_ *β*_7_ integrin and L-selectin. The sum varied between 105.5–149.4% thus suggesting that some cells were at least double positive, that is, expressed more than one HR. This phenomenon is suggested also by the height of the columns in Figure 3.

## 4. Discussion

Immune responses are not evenly distributed in the body, but, instead, activated lymphocytes are guided to those tissues where their specific antigen is expected to be encountered [13]. The immune system decides on these sites on the basis of where the new antigen was first encountered; each pathogen has a typical environmental locus and route of transmission. Consequently, upon new encounters, the pathogen tends to use the same route of invasion. Thus it is advantageous to concentrate the immune response at that site. In cancer vaccines, the effector cells should be guided to the area where the cancer has originated or spread; with mucosal cancers, a mucosal homing profile, and with nonmucosal cancers, a systemic homing profile appears as to be most advantageous. The present study describes the homing profile of the immune response to KLH, a protein carrier in numerous promising cancer vaccine candidates. These data should be applicable to all KLH-conjugated cancer vaccines. The effect of possible oral priming with KLH was explored at the same time.

Results from early clinical trials for idiotype vaccines suggested that both humoral and cellular immune responses may be independently associated with tumor regression and improved progression-free survival [7]. We have previously published data on KLH-specific T cell-mediated immunity [10, 19]; the homing profile was found to depend on the site of antigen encounter, oral or parenteral [19]. In the present study, KLH-specific B cell responses were examined in three different settings. Consistently with previous studies of B cells using the same feeding protocol for KLH [10], no response manifested by ASC was elicited by oral administration. However, in our previous studies [10, 19], it has been shown that KLH-specific T cells are found after oral KLH feeding, and, after a subsequent subcutaneous administration, the B cell response is enhanced as compared to a nonprimed group. The results of the present study appear to be consistent with those findings, as indicated by the geometric mean which appeared lower in the nonprimed than in the orally primed group (60 versus 136 ASC/10^6^ cells). The enhancement of B cell response resulting from preceding oral feeding appears to offer an opportunity of utilization for cancer vaccines. However, the simultaneously induced tolerance of T cells [10, 19] appears an undesired consequence which may interfere with the idea of using oral KLH priming as a means of enhancing immune response to conjugated cancer vaccines.

In both this study and the previous studies, KLH originated from two different sources: “Calbiochem KLH" for oral use versus “Pacific Biomarine KLH" for parenteral use. Although both preparations certainly contain the same protein, they differ in the grade of purity, possibly in the assembly state of the protein, isoform proportion and percentage of denatured versus native protein. Moreover, the oral preparation was probably contaminated by endotoxin. However, these factors presumably had no major impact on the results of the present study where the immune responses were measured with a highly purified KLH preparation, and the main focus was on the homing of the KLH-specific lymphocytes.

The homing of lymphocytes into various tissues is guided by the expression of HR and CCR on their surfaces [12, 13]. The combinations of these molecules act as trafficking programmes imprinted on them during activation, targeting their migration to specific tissues and microenvironments. Dendritic cells in the tissues present the antigen to the lymphocytes in the tissues, simultaneously providing instructions for the expression of HR and CCR, that is, the homing profile [12, 13]. Dendritic cells from different tissues give different instructions. The lymphocytes are generally guided to travel back to the site of antigen encounter, but possibly to certain other sites as well. Therefore, it would be useful to identify the trafficking patterns so as to be able to guide the cells to sites where they are desired. Paradoxically, the majority of vaccines currently used against microbes are given by systemic route, as injections at cutaneous sites, although the majority of microbial pathogens gain access to the body through the mucosal sites. With cancer vaccines, it appears that the cells should be targeted to the site where the tumour grows and its possible metastatic foci. In the present study, a systemic homing profile with high proportions of L-selectin^+^ ASC and low proportions of integrin *α*_4_ *β*_7_^+^ ASC was revealed in both nonprimed and primed groups. However, while L-selectin expressions were identical in the two groups, the proportion of *α*_*4*_ *β*_*7*_ integrin-expressing cells appeared higher in the primed than in nonprimed volunteers. An analogous increased proportion of *α*_4_ *β*_7_ integrin in orally primed volunteers after a subsequent subcutaneous booster immunization was found in our previous study on *Salmonella typhi* Ty21a vaccines [28]. These data suggest a slightly increased targeting to the intestine in the primed group. This change appears undesirable for vaccines against systemic cancers and adds to the negative effects of oral priming with KLH. 

The homing profile for KLH-conjugated cancer vaccines is likely to prove similar to the homing profile presented here for parenteral KLH. The vaccine was only given to healthy volunteers, no cancer patients were included. However, even if cancer patients with immunosuppression are expected to exhibit a less vigorous response, the disease will presumably have no significant impact on the homing profiles of the immune effector cells. The systemic homing profile for subcutaneous KLH indicated by the present study appears to be the desirable type of HR profile for responses against nonmucosal cancers at systemic sites, thus encouraging the development of this kind of parenterally administered vaccines.

## Abbreviations

ASC: antibody-secreting cell,
CCR: chemokine receptor,
HR: homing receptor,
ISC: immunoglobulin-secreting cell,
KLH: keyhole limpet haemocyanin.
